# An overview of machine learning methods for monotherapy drug response prediction

**DOI:** 10.1093/bib/bbab408

**Published:** 2021-10-08

**Authors:** Farzaneh Firoozbakht, Behnam Yousefi, Benno Schwikowski

**Affiliations:** Systems Biology Group, Department of Computational Biology, Institut Pasteur, Paris, France; Systems Biology Group, Department of Computational Biology, Institut Pasteur, Paris, France; Sorbonne Université, École Doctorale Complexite du Vivant, Paris, France; Systems Biology Group, Department of Computational Biology, Institut Pasteur, Paris, France

## Abstract

For an increasing number of preclinical samples, both detailed molecular profiles and their responses to various drugs are becoming available. Efforts to understand, and predict, drug responses in a data-driven manner have led to a proliferation of machine learning (ML) methods, with the longer term ambition of predicting clinical drug responses. Here, we provide a uniquely wide and deep systematic review of the rapidly evolving literature on monotherapy drug response prediction, with a systematic characterization and classification that comprises more than 70 ML methods in 13 subclasses, their input and output data types, modes of evaluation, and code and software availability. ML experts are provided with a fundamental understanding of the biological problem, and how ML methods are configured for it. Biologists and biomedical researchers are introduced to the basic principles of applicable ML methods, and their application to the problem of drug response prediction. We also provide systematic overviews of commonly used data sources used for training and evaluation methods.

## Introduction

The continued development of new high-throughput molecular profiling (‘omics’) technologies and their concomitant increased accessibility for biomedical applications make it increasingly attractive to study complex biological phenomena using data-driven and machine learning (ML) approaches [1]. ML is a specific subset of artificial intelligence that allows automatic learning from data, and has contributed to a wide range of genomics research [2]. One particular application of ML is drug response prediction (DRP), in which phenotypic responses of biological samples are predicted on the basis of their molecular profiles, with predictors often providing mechanistic insight.

In the context of biomedical research, DRP has, until now, largely focused on the response to anticancer drugs, possibly owing to the importance of cancer as a major public health issue, and the relative ease with which cancer cells can be cultured for *in vitro* drug screening assays. Cancer is a multifactorial and heterogeneous disease; each tumour typically has its own unique molecular characteristics, often leading to diverse responses to the same therapy for patients despite similar clinical phenotypes [3]. Hence, personalized, instead of one-size-fits-all, approaches to therapy are thought to have great potential to improve the clinical treatment of cancer [4] by extending survival and reducing side effects [5].

Unlike in other applications of ML, the establishment of systematic training datasets, e.g. in clinical trials, where a large number of drugs are evaluated for each of a large number of patients, is ethically impossible, and practically extremely expensive to approximate. The systematic datasets used for DRP therefore come mainly from preclinical models, such as cell lines [6], organoids [7] and xenograft mice [8]. In contrast to cancer cell lines, organoids and xenograft mice comprise interactions with the tumour microenvironment, and are thus considered to be better models for clinical outcomes [9–11]. Another concern with cell lines is that they may diverge from the original tumour. Yet, the observable pharmacological patterns in cell lines, which are relatively easy to culture, are still considered as rich in information about the drug mechanisms of action [6, 12, 13], and encompass clinically relevant genomic alterations [14]. The relatively good availability of large-scale, systematic, cell line DRP datasets [15–18] make them the most frequently used input for ML-based DRP.

Given the omics data acquired from cell lines or tumours, along with their phenotypic responses to drugs, various ML approaches have been employed for DRP, with different inputs, models and learning processes, and different levels of complexity and interpretability of the resulting DRP models. DRP models range from established simple linear regression models to recent and more complex such as deep neural networks (DNNs). In addition, DRP methods can use biomolecular networks (between, e.g. genes and cell lines) that represent known or derived relationships.

The most recent comprehensive review of DRP methods, published in 2015 [19], naturally does not encompass important recent developments, such as recommender systems-based DRP. Other, more recent, reviews focus on specific aspects. Adam *et al.* [20] provide a broad view of methods linked to recent technologies, such as deep learning and single cell sequencing, and discuss important extensions, such as combination therapy and the prediction of drug resistance. Ali and Aittokallio [21] focus on the principled lessons from the NCI-DREAM7 community challenge [22]. Paltun *et al.* [23] discuss three specific subclasses of DRP methods based on data integration approaches. Furthermore, Chen and Zhang [24] provide a systematic performance evaluation of 17 selected DRP methods.

The present review thus intends to provide a structured, up to date, panorama of DRP methods from the past 10 years, rather than deeper technical discussions relating to specific classes of methods, which can be found in some of the above reviews and the original and ML literature. While introducing basic biological and ML concepts when needed, (i) we discuss all major databases commonly used to learn DRP ML models, together with a systematic overview of the drug response measurement that was used, numbers of drugs/samples, and URLs, (ii) we survey more than 70 DRP methods across 13 subcategories, together with a systematic overview of used inputs, outputs, evaluation approach, and code and software availability, and (iii) we discuss common approaches to evaluate DRP methods.

## Databases

A number of ambitious experimental efforts have produced large *pharmacogenomic* datasets, i.e. collections of drug responses of clinical or preclinical samples, along with their molecular profiles. Box I provides an overview of commonly used preclinical drug response metrics that are used to represent the drug responses in these datasets.

Pharmacogenomic data have provided insight into the relationship between clinical and preclinical molecular profiles and responses to anticancer drugs, and drug response-predictive biomarkers. In this section, we survey the commonly used large publicly available pharmogenomic datasets.

The Cancer Genome Atlas (TCGA) [25] provides genomic, epigenomic, transcriptomic and proteomic features of over 11 000 patients across 33 cancer types. TCGA is a collaborative effort between the National Cancer Institute and the National Human Genome Research Institute started in 2006 aiming to better understand cancer biology and improve cancer treatment and prevention. In TCGA, patient drug responses are categorized into four classes of complete response, partial response, progressive disease and stable disease*,* based on tumour growth measured by RECIST, an imaging-based criterion to evaluate the response in solid tumours [26]. The TCGA dataset can be downloaded from Genomic Data Commons Data Portal.

Based on the hypothesis that *in vitro* drug activity on cancer cell lines can capture essential aspects of *in vivo* drug activity on corresponding tumours [12], molecular and drug sensitivity profiles across a large number of cell lines have been generated; Table 1 provides an overview. In the late 1980s, a project led by the US National Cancer Institute measured the ability of thousands of anticancer drugs to inhibit cell growth across 60 human cancer cell lines [6, 12]. The *NCI-60* panel of measurements includes gene expression (GE), copy number variation (CNV), single nucleotide polymorphism (SNP), DNA methylation (DM) and proteomics profiles of each cell line. Analysis of this data resulted in the discovery of a number of gene mutations that are highly predictive of clinical responses [27] and provided new insights into drug activity modulators and drug-target interactions.

**Table 1:** Publicly available drug screening datasets on cell lines

Datasets	Drug response measurement	Sample size	Drug number	Tissue number	Reference	URL
NCI60	GI50, IC50, LC50, TGI	60	<3000	9	Shoemaker *et al.* (2006)	https://dtp.cancer.gov/discovery_development/nci-60/
Genomics of Drug Sensitivity in Cancer (GDSC)	IC50, AUC	988	518	36	Garnett *et al.* (2012) Yang *et al.* (2012) Iorio *et al.* (2016)	https://www.cancerrxgene.org
Cancer Cell Line Encyclopedia (CCLE)	IC50, EC50, AA	504	24	36	Barretina *et al.* (2012)	https://portals.broadinstitute.org/ccle
GRAY	GI50	70	90	1	Heiser *et al.* (2012) Daemen *et al.* (2013)	https://link.springer.com/article/10.1186/gb-2013-14-10-r110#additional-information
Institute for Molecular Medicine Finland (FIMM)	EC50, DSS	106	308	1	Pemovska *et al.* (2013)	https://cancerdiscovery.aacrjournals.org/content/3/12/1416.figures-only
Genentech (GNE)	IC50	675	>350	17	Klijn *et al.* (2015)	https://www.nature.com/articles/nbt.3080#MOESM21
Human B-cell Cancer Cell Lines (HBCCL)	AUC, Sen/Res	26	3	2	Falgreen *et al.*, (2015)	https://www.ncbi.nlm.nih.gov/geo/query/acc.cgi?acc=GSE53798
Cancer Therapeutics Response Portal (CTRPv2)	AUC	860	481	25	Seashore-Ludlow *et al.* (2015)	http://portals.broadinstitute.org/ctrp.v2.1/
Genentech Cell Line Screening Initiative (gCSI)	AUC, IC50	410	16	23	Haverty *et al.* (2016)	https://pharmacodb.pmgenomics.ca/datasets/4
Head and neck squamous cell carcinomas cell lines	IC50	8	276	1	Chia *et al.* (2017)	https://www.chip-phenomics.org/DOWNLOAD/Chia2017/
Texas Southwestern Medical Center non-small cell lung cancer cell line	AUC, ED50	100	222	2	McMillan *et al.* (2018)	https://www.ncbi.nlm.nih.gov/geo/query/acc.cgi?acc=GSE104757

Following the NCI-60 effort, two large collaborative datasets were established: the Cancer Cell Line Encyclopedia (CCLE) [16] and the Genomics of Drug Sensitivity in Cancer (GDSC) [15]. CCLE is a collaboration of the Broad Institute and Novartis, and GDSC is a collaboration between The Cancer Genome Project at the Wellcome Trust Sanger Institute (UK) and the Center for Molecular Therapeutics at the Massachusetts General Hospital Cancer Center (USA). Both datasets contain GE, CNV, DM, and mutational status (MS) of around 1000 human cancer cell lines from various tumour types, together with pharmacological profiles across a large fraction of these cell lines, for 24 and 518 drugs, respectively, in CCLE and GDSC. Although several studies pointed at inconsistencies in the drug response measurements between CCLE and GDSC [28, 29], others argued that the inconsistencies disappear under a specific, reasonable, interpretation of the data [18, 30–33]. To shed more light on this issue, the Genentech Cell Line Screening Initiative [18] generated profiled 410 cell lines common to GDSC and CCLE using various approaches, including RNA-seq GE, MS and CNV, along with their responses to 16 drugs tested in both studies. The outcomes of this study supported the previously proposed mode of consistent interpretability across CCLE and GDSC data. PharmacoDB [34] provides large publicly available preclinical datasets that have been prepared for integrated use, with special emphasis on consistency.

The Cancer Therapeutics Response Portal (CTRP) [17] provided another pharmacogenomic dataset with a variety of different molecular features of cell lines, including MS, GE, CNV and tissue type. The last published version of this dataset includes 860 characterized cancer cell lines and their responses to 481 compounds. DepMap is a data repository that aggregates a number of different preclinical datasets (including GDSC, CCLE and CTRP).

## DRP models

**Figure 1:**
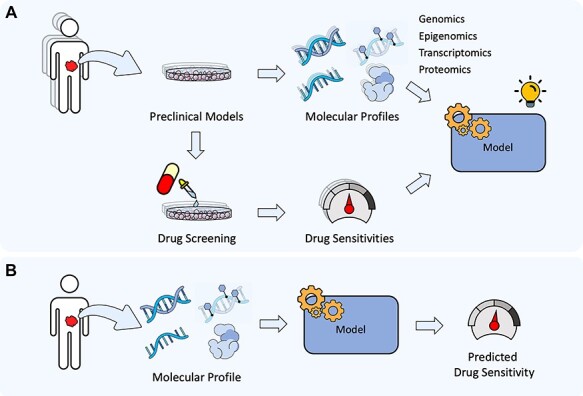
(A) Preclinical samples (e.g. cell lines), on one hand, are profiled to generate omics data, and on the other hand, they are screened over different drugs for obtaining their sensitivities. This information together is used to train machine learning models. (b) The trained model predicts the sensitivity for a new cell line.

The high complexity of the biological response to drugs and the increasing feasibility of large-scale molecular profiling experiments make ML approaches increasingly attractive for modelling and prediction of drug responses. DRP models are most often established using a set of known phenotypic drug responses (supervised learning). Generally, the objective of DRP models is the prediction of the response of drugs for a given sample from its molecular profile (Figure 1). Regression estimates a quantitative drug response (Box I) for each sample, while classification predicts discrete labels that stratify samples into sensitive or resistant. To obtain ground truth labels in preclinical samples, the continuous measure of drug response is then thresholded by methods such as median-based [35], maximum concentration-based [14], waterfall-based [16] and predefined [36–38]. From a practical perspective, discretization enables the use of classification methods, but it also loses information, and, if predictions are to be interpreted in a clinical context, the clinical relevance of the resulting classes requires special attention.

Feature selection, i.e. the maximal removal of data features with a minimal loss of problem-relevant information, is an important part of DRP, as it is a major antidote to the statistical and computational problems that the high-dimensional omics input data typically entail. For focused discussions of feature selection, we refer the reader to Webb and Copsey [39], and, for their use in DRP, to the study by Koras *et al.* [40].

Network-based machine learning models are relatively recent, and increasingly popular for DRP. Here, networks (often equivalent to mathematical graphs) can refer to sets of interactions between cell lines, drugs, genes, proteins or their combination. Networks carry different types of information, and their use and interpretation vary greatly across the different uses for DRP.

DRP models can be classified as single-drug learning (SDL) and multi-drug learning (MDL). To predict the response to a given drug, MDL leverages data from other drugs; SDL exclusively uses data about the given drug. SDL and MDL differ in their capabilities and validation process. Table 2 provides a systematic overview of all DRP articles covered here.

### SDL models

SDL models are trained to predict drug responses for a given drug, independent of other drugs. In this section, we survey the SDL approaches in the literature across seven subclasses.

#### Linear regression

Regression analysis is a set of statistical methods for estimating the relationship between a dependent variable and one or more independent variables. As its simplest form, linear regression uses a linear function to model this relationship, and enjoys remarkable popularity due to its simplicity and interpretability.

In DRP, the number of available observations often does not suffice to determine a unique model. Regularization techniques address this issue by limiting the possible solutions through additional assumptions [41, 42], and are therefore a popular choice for DRP. Shrinkage is a form of regularization in which parameter values close to zero are preferred when they do not clearly contribute to the predictive performance of the model. Ridge regression [43] includes a form of shrinkage, and was used to assess whether models trained on preclinical samples can predict clinical drug responses [30, 44]. Ridge regression typically does not yield sparse models (i.e. models with few independent variables that are easier to interpret). Lasso regularization, on the other hand, forces coefficients to zero, their number being controlled by a hyperparameter [45]. In a recent study, Huang *et al.* proposed Tissue-Guided Lasso to derive tissue-specific models through tissue-specific choices for their hyperparameter. They defined tissue-specific hyperparameters by training each lasso model on all cell lines, excluding those from the tissue of interest, and then calibrated the hyperparameter using data from that tissue [46]. One principled limitation of Lasso is that the number of non-zero coefficients cannot be higher than the number of data samples [42]. To derive sparse models even in cases when the number of features is larger than the sample size, the elastic net [41] combines ridge regression and lasso regularization, and has been also used for DRP [16, 47–50].

Biomolecular networks represent interactions of biomolecules in the same functional context that can be leveraged for DRP. These interactions can be derived from prior knowledge or independent experimental data. A common use of networks for DRP is to use them for regularization, by assuming that model coefficients for functionally related molecules are similar. Sokolov *et al.* [51] introduced the generalized elastic net model that incorporates the Pathway Commons [52] network in an elastic net approach through a network-based regularization term.

Partial least squares regression (PLSR) is another type of linear method, based on latent variable projection [53]. In PLSR, input and output data are each projected into a (usually low-dimensional) new space, and then a relationship between them is established using linear regression. This makes PLSR suitable for analysing high-dimensional data and DRP [35, 54].

#### Logistic regression

Instead of predicting drug response as a continuous variable, samples can also be classified as sensitive or resistant. Logistic regression is a common classification approach that can be derived, in a Bayesian framework, from a simple probabilistic model that expresses drug response as a function of molecular profiles [30, 44, 55, 56]. Classifiers derived from these models have linear decision boundaries, i.e. lines or hyperplanes that separate different classes in the input space [42, 57]. The assumption of linearity can be a strength in the case of small datasets, but a limitation when larger datasets would be able to resolve more complex decision boundaries. Huang *et al.* [58] proposed a logistic regression model that is additionally constrained based on biomolecular networks.

**Table 2:** Systematic overview of drug response prediction methods

Model	Paper	Method name	Dataset	Input	Output	Evaluation	Code/software availability
**Single-drug learning (SDL) models**							
Linear Regression							
	Barretina *et al.* (2012)	-	CCLE	MAGE, CNV, Mu, TT	IC50, AA	KFCV	-
	Papillon-Cavanagh *et al.* (2013)	-	GDSC, CCLE	MAGE	IC50	KFCV, CDV	-
	Niepel *et al.* (2013)	-	NCI	Mu	GI50	LOOCV	-
	Geeleher *et al.* (2014)	-	GDSC, three clinical datasets	MAGE	IC50	LOOCV, CDV	http://geeleherlab.org/cgpPrediction/
	Falgreen *et al.* (2015)	-	HBCCL, GDSC, IDRC, LLMPP, MDFCI, UAMS	MAGE	AUC	KFCV, LOOCV	-
	Covell *et al.* (2015)	-	GDSC, CCLE	MAGE	GI50	CDV	-
	Li *et al.* (2015)	-	OncoPanel, BATTLE	MAGE	IC50	RRSSCV	-
	Sokolov *et al.* (2016)	GELnet	LBNLBC	MAGE	GI50	LPOCV	https://cran.r-project.org/web/packages/gelnet/index.html
	Aben *et al.* (2016)	TANDEM	GDSC	MAGE, Mu, CNV, DM, TT	IC50	KFCV	https://cran.r-project.org/web/packages/TANDEM/index.html
	Huang *et al.* (2020)	TG-LASSO	GDSC, TCGA	MAGE	IC50	RRSSCV, CDV	https://github.com/emad2/TG-LASSO
Logistic Regression							
	Geeleher *et al.* (2014)	-	GDSC, three clinical datasets	MAGE	Res/Non-Res	LOOCV, CDV	http://geeleherlab.org/cgpPrediction/
	Falgreen *et al.* (2015)	-	HBCCL, GDSC, IDRC, LLMPP, MDFCI, UAMS	MAGE	Sen/Res	KFCV, LOOCV	-
	Ding *et al.* (2016)	-	TCGA	MAGE, CNV, DM, miRNA	Sen/Res		-
	Geeleher *et al.* (2017)	-	GDSC, TCGA	MAGE	IC50	KFCV, CDV	https://osf.io/pwm4z/
	Ding *et al.* (2018)	-	GDSC, CCLE	MAGE, CNV, Mu	Sen/Res	RRSSCV	-
	Huang *et al.* (2018)	L*q*-NLR	GDSC, BATTLE	MAGE	IC50	KFCV	-
Maximum Margin Models							
	Dong *et al.* (2015)	-	GDSC, CCLE	MAGE	Sen/Res	KFCV, CDV	-
	Gupta *et al.* (2016)	-	CCLE	MAGE, CNV, Mu	IC50	-	http://crdd.osdd.net/raghava/cancerdp/
	Parca *et al.* (2019)	-	GDSC	MAGE	IC50	KFCV	https://github.com/lucaparca/dre
Ensemble Learning Methods							
	Riddick *et al.* (2010)	-	NCI-60	MAGE	IC50	OOBP	-
	Daemen *et al.* (2013)	-	LBNLBC, TCGA	MAGE, DM, ES, RSGE, PA, CNV	Sen/Res	RRSSCV, CDV	-
	Stetson *et al.* (2014)	-	NCI60, CCLE, GDSC	MAGE, SNP, CNV	IC50	KFCV, CDV	-
	Wan & Pal (2014)	-	CCLE, NCI-DREAM	MAGE, CNV, PA, DM, RSGE	GI50, IC50	KFCV, LOOCV	-
	Fang *et al.* (2018)	QRF	CCLE	MAGE, Mu, CNV	AA	OOBP	-
	Xu *et al.* (2019)	AutoBorutaRF	GDSC, CCLE	MAGE, SNP, CNV	Sen/Res	KFCV	https://github.com/bioinformatics-xu/AutoBorutaRF
	Oskooei *et al.* (2019)	NetBiTE	GDSC	MAGE	IC50	KFCV	-
	Su *et al.* (2019)	Deep-Resp-Forest	GDSC, CCLE	MAGE, CNV	Sen/Res	KFCV	https://github.com/RanSuLab/Deep-Resp-Forest
	Kurilov *et al.* (2020)	-	GDSC, CCLE, CTRP, gCSI, NIBR PDXE	MAGE, CNV, Mu, RSGE, TT	IC50, AUC, V1, TVC	RRSSCV	https://github.com/RomaHD/DrugRespPrediction
Nearest Neighbour Method							
	Li *et al.* (2021)	GA/KNN	GDSC, CCLE, TCGA, GTEx	MAGE, RSGE	IC50	RRSSCV, CDV	-
Artificial Neural Networks and Deep Learning							
	Sakellaropoulos *et al.* (2019)	-	GDSC, TCGA, MD Anderson, OCCAMS, multiple myeloma	MAGE	IC50	KFCV	https://github.com/TeoSakel/deep-drug-response
	Sharifi-Noghabi *et al.* (2019)	MOLI	GDSC, PDX, TCGA	MAGE, SNP, CNV	Res/Non-Res	CDV	https://github.com/hosseinshn/MOLI
	Ahmed *et al.* (2020)	-	NSCLCCLP	RSGE	AUC, ED50	RRSSCV	https://github.com/compbiolabucf/drug-sensitivity-prediction
	Ma *et al.* (2021)	TCRP	GDSC, CCLE, DepMap, PDTC BioBank, PDX Encyclopedia	MAGE, Mu	AUC, TVC	CDV	https://github.com/idekerlab/TCRP/
	Malik *et al.* (2021)	-	GDSC, TCGA	MAGE, Mu, CNV, DM	IC50	KFCV, CDV	https://github.com/TeamSundar/BRCA_multiomics
Molecular Network Similarity-Based Methods							
	Kim *et al.* (2016)	NBC	GDSC, CCLE	MAGE	Sen/Res	KFCV	-
	Stanfield *et al.* (2017)	-	GDSC, CCLE	SNP	CDSS	LOOCV, CDV	-
**Multi-drug learning (MDL) models**							
Linear Regression							
	Costello *et al.* (2014)	Bayesian multitask MKL	NCI-DREAM, GDSC	MAGE, RSGE, CNV, Mu, DM, PA	GI50	challenge test cell lines	https://www.nature.com/articles/nbt.2877#MOESM3
	Yuan *et al.* (2016)	-	CCLE, CTRPv2, NCI60	MAGE, CNV, Mu	AA, AUC, GI50	KFCV	-
	Ammad-ud-din *et al.* (2017)	MVLR	GDSC, FIMM	MAGE	IC50, CDSS	LOOCV	https://github.com/suleimank/mvlr
Ensemble Learning Methods							
	Matlock *et al.* (2018)	-	GDSC, CCLE	MAGE, DT, DD	AUC	RRSSCV	-
	Liu *et al.* (2020)	-	GDSC, CCLE	MAGE	AA, AUC	KFCV	https://zenodo.org/record/1325121#.YNHB_i0RozV
	Sharma & Rani (2019)	-	GDSC, CCLE, NCI-Dream	MAGE	IC50, GI50	KFCV	-
	Su *et al.* (2020)	Meta-GDBP	GDSC, CCLE	MAGE, DD	IC50, AA	KFCV, CDV	https://github.com/RanSuLab/Meta-GDBP
Artificial Neural Networks and Deep Learning							
	Menden *et al.* (2013)	-	GDSC	MS, MU, CNV, DD	IC50	KFCV	-
	Chang *et al.* (2018)	CDRscan	GDSC, CCLP	SNP, DD	IC50	KFCV	-
	Li *et al.* (2019)	Deep DSC	GDSC, CCLE	MAGE, DD	IC50	KFCV, LOTO, LOCO	-
	Chiu *et al.* (2019)	DeepDR	GDSC, TCGA	MAGE, Mu	IC50	RRSSCV	-
	Joo *et al.* (2019)	DeepIC50	GDSC, CCLE, TCGA	Mu, DD	3-class sensitivity	RRSSCV, CDV	https://github.com/labnams/DeepIC50
	Liu *et al.* (2019)	tCNNS	GDSC	Mu, CNV, DD	IC50	RRSSCV, LOTO	https://github.com/Lowpassfilter/tCNNS-Project
	Manica *et al.* (2019)	MCA	GDSC	MAGE, DD	IC50	KFCV, RRSSCV	https://github.com/drugilsberg/paccmann https://ibm.biz/paccmann-aas
	Choi *et al.* (2020)	RefDNN	GDSC, CCLE	MAGE, DD	Sen/Res	KFCV	https://github.com/mathcom/RefDNN
	Zhu *et al.* (2020)	-	GDSC, CCLE, CTRP, gCSI	MAGE, DD	AUC	KFCC	-
	Bazgir *et al.* (2020)	REFINED	GDSC, NCI-60	MAGE, DD	IC50, GI50, Sen/Res	KFCV, RRSSCV	https://github.com/omidbazgirTTU/REFINED
	Liu *et al.* (2020)	DeepCDR	GDSC, CCLE, TCGA	MAGE, Mu, DM, DD	IC50, Sen/Res	KFCV, CDV	https://github.com/kimmo1019/DeepCDR
	Tang & Gottlieb (2021)	PathDSP	GDSC, CCLE	MAGE, CNV, Mu, DD	IC50	KFCV, CDV	https://github.com/TangYiChing/PathDSP
	Nguyen *et al.* (2021)	GraphDRP	GDSC	Mu, CNV, DD	IC50	RRSSCV	https://github.com/hauldhut/GraphDRP
Recommender Systems (neighbourhood-based)							
	Zhang *et al.* (2015)	Dual-layer integrated cell line-drug network	GDSC, CCLE	MAGE, DD	AA, IC50	LOOCV	-
	Sheng *et al.* (2015)	-	GDSC, CCLE	MAGE, DD	IC50	KFCV, CDV	-
	Liu *et al.* (2018)	NCFGER	GDSC, CCLE	MAGE, DD	IC50, AA	KFCV	-
	Zhang *et al.* (2018)	HIWCF	GDSC, CCLE	MAGE, DD, DT	IC50, AA	KFCV	https://github.com/laureniezhang/HIWCF
	Le & Pham (2018)	GloNetDRP	GDSC, CCLE	MAGE, Mu	AUC, IC50	KFCV	-
	Wei *et al.* (2019)	CDCN	GDSC, CCLE	MAGE, DD	AA, Sen/Res	LOOCV	https://zenodo.org/record/1403638#.YNHVyi0RozV
Recommender Systems (model-based)							
	Ammad-ud-din *et al.* (2014)	KBMF	GDSC	MAGE, CNV, Mu, DD, DT	IC50	KFCV	https://research.cs.aalto.fi/pml/software/kbmf/
	Ammad-ud-din *et al.* (2016)	cwKBMF	GDSC, CTRPv1, AMLCLP	MAGE, DD	IC50, AUC	KFCV, CDV	https://research.cs.aalto.fi/pml/software/cwkbmf/
	Wang *et al.* (2017)	SRMF	GDSC, CCLE	MAGE, DD	AA, IC50	KFCV	https://github.com/linwang1982/SRMF
	Suphavilai *et al.* (2018)	CaDRReS	GDSC, CCLE, HNCCLP	MAGE	IC50	KFCV	https://github.com/CSB5/CaDRReS
	Yang *et al.* (2018)	Macau	GDSC	MAGE, DT	IC50	KFCV	https://github.com/saezlab/Macau_project_1
	Guan *et al.* (2019)	WGRMF	GDSC, CCLE	MAGE, DD	IC50, AA	KFCV	-
	Moughari & Eslahchi (2020)	ADRML	GDSC, CCLE	MAGE, Mu, CNV, DD, DT	IC50	KFCV	https://github.com/fahmadimoughari/ADRML
	Emdadi & Eslahchi (2020)	DSPLMF	GDSC, CCLE	MAGE, Mu, CNV, DD	Sen/Res	KFCV	https://github.com/emdadi/DSPLMF
	Emdadi & Eslahchi (2021)	Auto-HMM-LMF	GDSC, CCLE	MAGE, CNV, Mu, TT, DD	Sen/Res	KFCV	https://github.com/emdadi/Auto-HMM-LMF
Network Representation Learning-Based Models							
	Yang *et al.* (2019)	NRL2DRP	GDSC	Mu, CNV, DM	Sen/Res	KFCV	https://github.com/USTC-HIlab/NRL2DRP
	Yu *et al.* (2020)	DREMO	GDSC, CCLE	MAGE, Mu, CNV, DD, DT	Sen/Res	KFCV	https://github.com/LiangYu-Xidian/MNDRP
Network Propagation-Based Method	Zhang *et al.* (2018)	HNMDRP	GDSC	MAGE, DD	Sen/Res	LOOCV	https://github.com/USTC-HIlab/HNMDRP

AA = activity area; AUC = area under curve; CDSS = custom drug sensitivity score; CDV = cross-dataset validation; CNV = copy number variation; DD = drug descriptors; DM = DNA methylation; ES = exome sequencing; MAGE = microarray gene expression; KFCV = K-fold cross validation; LOCO = leave-one-compound-out; LOOCV = leave-one-out cross validation; LOTO = leave-one-tissue-out; LPOCV = leave-pair-out cross validation; miRNA = micro-RNA; MS = microsatellite; Mu = mutation; OOBP = out-of-bag prediction; PA = protein abundance; Res/Non-Res = responder/non-responder; RSGE = RNA-seq gene expression; RRSSCV = repeated random sub-sampling cross validation; Sen/Res = sensitive/resistance; SNP = single nucleotide polymorphism; TT = tissue type; TVC = tumor volume changes; V1 = viability at 1 μm.

#### Support vector machines

Support vector machines (SVMs) [42, 57, 59] are ML models that can be used for classification and regression. SVMs are efficiently learnable, and can employ nonlinear kernels (nonlinear transformation functions). Dong *et al.* used a SVM to classify samples as sensitive and resistant. SVM regression (SVR) [60] has also been used for drug prioritization [61] and response prediction [62, 63]. The selection of a kernel function and the interpretation of SVMs are typically not straightforward.

#### Nearest neighbour method

Nearest neighbour methods are simple and intuitively understandable classification and regression methods. In the *K-*Nearest Neighbour (KNN) method, the output for each new sample is on the basis of the outputs of a smaller number (commonly denoted by *K*) of molecular profiles that are the most similar to the profile of the new sample [42]. Li *et al.* [64] employed KNN regression for DRP, in which the sensitivity of a new sample is modelled as the average of the observed sensitivity values of its KNNs. Nearest neighbour methods tend to be problematic in the inadvertent, and typically unknown, presence of many irrelevant data features, and they are computationally intensive.

#### Artificial neural networks and deep learning

**Figure 2:**
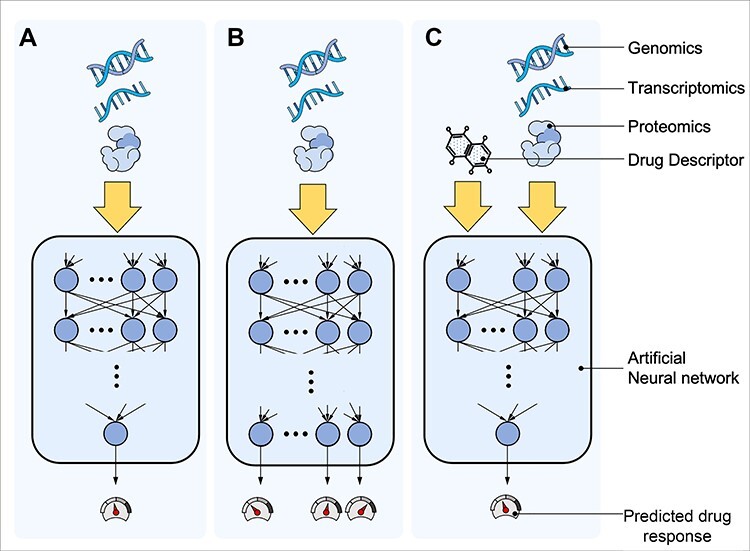
Artificial neural networks in drug response prediction can be configured as (A) single drug learning (SDL) or (B, C) multi-drug learning (MDL). SDL ANNs learns the response of a single drug for a sample using omics data, whereas MDL ANN learns the response to multiple drugs, which can have (B) multiple outputs, such that each output corresponds to the prediction for a specific drug, or (C) single output with the additional input of drug features.

Artificial neural networks (ANNs) are powerful ML models that can approximate arbitrary input–output relationships, given a large and diverse enough set of training samples. Multilayer feedforward neural networks are one of the most commonly used ANNs that consist of multiple layers [57]; each layer consists of a number of computational units, called neurons (Figure 2A). These ANNs are usually fully connected, i.e. the first layer of neurons receives the input data, and the outputs of all neurons in any given layer are used as inputs for all neurons in the next layer.

In their generic form, ANNs require more input data than simpler models. Ma *et al.* [65] used a transfer learning approach to address the problem of relatively little available DRP data for advanced preclinical models. In a first pre-training phase of their TCRP method, a multilayer ANN is learned using a larger number of basic preclinical training samples (cell lines). In the following learning phase, a small dataset from advanced preclinical models is used to recalibrate the model to the new context.

ANNs with many layers are called DNNs. Deep learning has recently attracted considerable attention across many fields and has become popular for DRP as well [66–68]. Encoders are particular types of ANNs, typically used in DNNs for feature reduction, i.e. the encoding of higher dimensional information using fewer dimensions with no or little loss of information. Stacked encoders–decoder networks can be trained simultaneously as autoencoders without the need for output data [69, 70]. Ding *et al.* trained a four-layer autoencoder, and the output of each layer was separately used to train a logistic elastic net to predict drug responses [56]. Sharifi-Noghabi *et al.* [71] introduced a DNN that learns encoders of different molecular data types separately, and whose outputs are fed to a multilayer neural network for DRP. Determining the best structure of ANNs and interpreting the resulting models are non-trivial, and learning ANNs tends to be computationally expensive.

Graph neural networks (GNNs) are ANNs that operate on graphs (i.e. sets of interactions) as inputs. Recently, deep GNNs have been used to learn low-dimensional representations of biomolecular networks [72, 73]. Ahmed *et al.* [74] employed two different GNN methods to use GE and a gene co-expression network, i.e. a network representing the correlation between the expression of gene pairs, to construct a GNN. In contrast to the use of GNNs for MDL (discussed in Section ANNs and Deep Learning below), GNNs have not been shown to improve significantly over the SDL performance of classical ML models.

#### Network similarity-based methods

Network similarity-based methods are based on the assumption that cell lines with similar network-level information respond similarly to the same drug. Class-specific networks, typically a sensitive network and a resistant network are then constructed to capture features characteristic for the samples from both classes. Although the above assumption underlying this relatively recent class of methods is simple, its validity needs to be established for each particular interpretation of network similarity.

Stanfield *et al.* [75] construct a ‘base network’ of genes and cell lines, in which cell lines are linked to their mutated genes, which, in turn, are connected by BioGRID protein–protein interactions [76]. To inject information about drug responses, the base network is then augmented with links between cell lines and the drug they are sensitive to, to obtain a sensitive network, and, analogously, a resistant network. The augmentation is designed to lead to tighter network connectivity between the drug, cell lines and genes involved in sensitivity or resistance, respectively. A new cell line is then classified based on the similarity of the connectivity of its base network with each of the class-specific networks. Kim *et al.* [77] proposed another biomolecular network-based classifier that exploits the presence of class-specific gene co-expression patterns. First, sensitive and resistant gene co-expression networks are constructed from the GE values of the corresponding cell lines. Next, for each gene in each of these networks, class-specific SVR models are trained to predict GE values on the basis of the GE of neighbouring genes in the class-specific network. For a new sample, the accuracy of the class-specific SVR prediction for any given gene is then evaluated, and interpreted as evidence for, or against, the membership of the new sample in the corresponding class.

#### Ensemble learning methods

By aggregating the predictions of multiple models, ensemble learning methods are often able to improve over the performance of any individual model [78]. Random forests (RFs) are established ensemble learning models that aggregate several regression or classification trees [79]. Trees are nonlinear ML models that predict the output by partitioning the input space into a hierarchical set of rectangles in the input space. The predicted output for the classification problem is the class label associated with the rectangle that corresponds to a new input. For the regression problem, the output is commonly the average value of the training outputs corresponding to each rectangle. RF trees are constructed from a random selection of the samples, and the remaining samples, called out-of-bag *samples*, can be used for validation. Riddick *et al.* [80] and Daemen *et al.* [81] initially used RF to predict drug sensitivity and identify molecular features associated with drug responses. Stetson *et al.* [82] selected differentially expressed genes between the most sensitive and resistant cell lines, and then used to train three classifiers using RF, SVM and logistic elastic net. Wan & Pal [83] proposed a RF regression-based approach using multiple omics data. To this end, they trained multiple RF regressions for different data types, and combined the individual predictions using linear regression. Moreover, Xu *et al.* [84] proposed a method called *AutoBorutaRF*, which employs an autoencoder-based feature selection algorithm and the Boruta algorithm [85] to improve the performance of RF.

To incorporate biological prior knowledge into the RF, Oskooei *et al.* [86] presented *NetBiTE*, in which a RF is biased towards using the known drug targets and their STRING PPI [87] interactors based on a network diffusion algorithm, i.e. a mathematical transformation of omics data that allows the detection of hypothetically relevant network regions [88]. Zhou & Feng [89] proposed an ensemble method called gcForest, whose performance was found to be competitive with DNNs. A modified version of gcForest, was used for drug response classification [38].

Fang *et al.* [90] argue that, due to the uncertainty inherent in DRP prediction, clinical applications benefit from richer output than a single predicted drug response. Their RF-based predictor is adapted to non-standard output distributions, and yields prediction intervals that, as they argue, may provide a better basis for the choice of appropriate treatments.

### MDL models

MDL models use data from multiple drugs to predict the response of a given drug. This may lead to improved predictions for drugs with training data, and, potentially, the prediction of responses to drugs for which no training data are available.

#### Multitask linear regression

Linear regression is also employed for MDL. In a DRP challenge competition performed by NCI-DREAM [22] the best-performing method was Bayesian multi-task multiple kernel learning (MKL), in which multiple data types (including original omics datasets and computationally derived data) are first kernelized (i.e. converted into nonlinear transfer functions), and then a global similarity matrix is constructed as their weighted sum. Next, a set of drug-specific weights, determined through multitask linear regression, are used to provide the final drug response values. Ammad-ud-din *et al.* [91] pointed out that, although kernels may enhance the model predictions, the resulting models are typically hard to interpret, in terms of the relationship of their inputs to their outputs. To address this issue, they created the *MVLR model*, which aggregates groups of molecular features that are known to be biologically connected, and use them as inputs to a multitask linear regression. In another study, Yuan *et al.* [92] jointly trained multilinear regression models based on trace norm regularization, which can increase the dependencies between model parameters to yield a more manageable number of biologically meaningful features.

#### ANNs and deep learning

ANNs are widely used in MDL. Through shared model parameters, MDL models can leverage information between different drugs and reduce the problem of overfitting, i.e. the learning of parameters driven by characteristics of the training data that are not necessarily present in other data. MDL ANNs have either single or multiple outputs. Each output of a multi-output *ANN* predicts the response for a specific drug (Figure 2B). Chiu *et al.* [93] proposed a multitask DNN model for DRP called *DeepDR*. Their method is composed of mutation and GE encoders that reduce data dimensionality, followed by a multilayer ANN with multiple outputs.

Single-output ANNs, on the other hand, accept omics data along with drug features as input (e.g. chemical descriptors and biological targets; Figure 2C). Unlike multi-output ANNs, they share all model parameters among multiple drugs. The potential advantage of single-output ANNs is their ability to provide predictions for new drugs whose data have not been used during the training phase. Menden *et al.* [94] employed a fully connected ANN to predict drug response. *DeepDSC* [95] first reduces the dimension of GEs using an encoder whose output is combined with the chemical drug features to be fed to a multilayer neural network. *RefDNN* [96] trains, for each drug, an SDL logistic elastic net regression model that predicts the probability of drug resistance. To predict the resistance of a given cell line to a new query drug, the vector of predicted resistance probabilities is then weighted by a structure similarity profile of the query drug with all the drugs as an input to a neural network to classify the response as sensitive or resistant. In an effort to leverage the observed activity of signalling pathways, Tang & Gottlieb [97] proposed a pathway-based method named *PathDSP*. In their approach, drug structure data along with the pathways enriched for drug targets, GE, somatic mutation and CNV are fed as inputs to a DNN. Zhu *et al.* [98] used transfer learning to overcome the challenge of learning with limited amounts of data by pre-training their model on a dataset from a related context, and then finalizing the training process in the context in which the model is designed to predict.

Convolutional neural networks (CNNs) are specific types of non-fully connected feedforward ANNs that can exploit natural adjacency structures in the data (such as physical or temporal adjacency) to more efficiently detect global patterns in the input data. CNNs work well for the inputs that contain spatial or temporal patterns (such as images) [70, 99, 100]. Joo *et al.* [101] proposed a CNN method, called *DeepIC50*, to predict three classes of high, intermediate and low responsiveness based on mutation status and drug descriptors. Moreover, in *CDRscan* [102], the mutation data of cell lines and the molecular fingerprints of drugs are fed to five different versions of a CNN, whose average predicted output value is reported as the final output. Liu *et al.* [103] introduced a model named *tCNNS*, in which two CNN branches are respectively used for cell line and drug features to predict drug sensitivities. Bazgir *et al.* [104] proposed a method called *REFINED* to convert GE profiles to an image that encodes relevant patterns of the molecular features in specific regions. The resulting images are then fed to a CNN for DRP.

GNNs can be used to encode drug features as points in a low-dimensional space, in which similar drugs are close to each other, by representing molecular structure of compounds as networks formed by sets of chemical bonds between atoms [105, 106]. In *DeepCDR* [107] and *GraphDRP* [108], deep drug features learnt by GNNs are concatenated with deep multi-omics features, to be fed to feedforward ANNs for DRP. Manica *et al.* [109] proposed the *MCA* approach, in which genes are selected based on a network diffusion algorithm [86]*.* Attention-based convolutional encoders are a recent new development from the field of natural language processing that has been useful to reduce the dimensionality of the GE and drug chemical data, to be used as input to a fully connected neural network for DRP [110].

#### Recommender systems

Recommender systems (RS) are algorithms that yield a set of personalized item suggestions to a user. RS were developed in the 1990s to automatically recommend movies and other products to users, based on their similarity of previously observed choices by the same user, or to the choices of other users with similar preferences [111, 112]. When transposed to DRP, this principle implies that a cell line is highly sensitive to a drug when similar cell lines are highly sensitive to similar drugs. Generally, RSs can be neighbourhood based or model based [111]. The main input used for the RS-based DRP models is a cell line-drug sensitivity matrix of sensitivity values of cell lines to the screened drugs. Complementary information, such as cell line molecular features and drug descriptor features, can also be used since they can enhance the similarity between different cell lines or different drugs.

##### Neighbourhood-based RS

**Figure 3:**
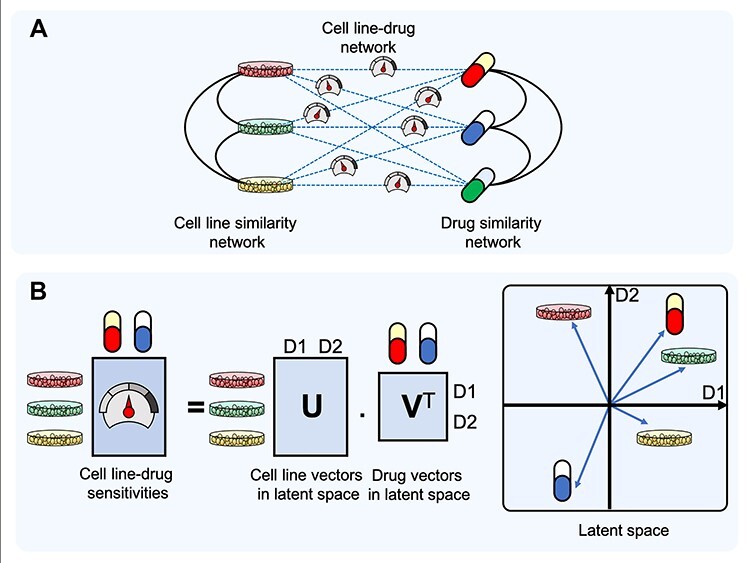
Two principal types of recommender systems (RS) for drug response prediction. (A) Neighbourhood-based RSs, which use cell line similarity and drug similarity measures to predict the response of a cell line to a drug. (B) Model-based RSs for DRP, which typically use matrix factorization (*left*), in which the cell line and drugs are represented as vectors in a *latent space* (*right*). The response of a cell line to a drug is then modelled to be proportional to the length of each of the vectors, and a decreasing function of the angle between cell line vector and the drug vector.

Neighbourhood-based methods (also called memory-based methods) are simple types of RS, in which the cell line-drug sensitivity prediction is based on the sensitivities of cell lines with similar characteristics to similar drugs, on the basis of cell line similarity network and drug similarity networks (Figure 3A) [113].

Zhang *et al.* [114] constructed cell line similarity and drug similarity networks using pairwise correlation of GEs and pairwise correlation of quantitative drug features, respectively. For a given cell line-drug pair, the predicted response is based on a weighted average of the known responses of cell lines to the given drug and all the drugs to the given cell line. These weights are normalized versions of cell line similarities and drug similarities. Le and Pham [115] tried to improve on this by basing their prediction upon all input cell line-drug responses.

One important drawback of the above approaches is their inability to predict in the case that drugs and cell lines are new (i.e. not part of the training data). To address this issue, Wei *et al.* [116] proposed an extension of Zhang’s method called *CDCN*. Conversely, Sheng *et al.* [117] raised the possibility that distinct drug responses between two cell lines might only manifest as GE differences in a few specific genes directly related to the drug resistance. To take this into account, they defined cell line similarity scores based on drug-specific sets of genes. To identify this set of genes for any given drug, their method first identifies drugs that are similar in terms of their chemical structures. Second, for each of those similar drugs, those genes whose GEs are highly associated with drug response are determined. Third, the similarity of all other cell lines to the given cell line is defined, based on the expressions of those genes. Finally, a similarity-weighted average of the IC50 values observed in other cell lines, based on the drug and cell line similarities, is used to predict the most sensitive drugs for the given cell line.

The cell line similarity and drug similarity measures in the above studies are only based on the molecular features of cell lines and drug descriptors, respectively. Conversely, the observed cell line-drug responses can also be used to compute meaningful similarity measures [118, 119].

Overall, neighbourhood-based RSs yield results based on a simple and intuitive principle. However, these methods tend to be heuristic in nature, due to their, typically ad hoc, selection of similarity measures and their subsequent heuristic downstream use. Further drawbacks of these methods are their typically high memory consumption and computational cost for testing samples, as well their difficult application on sparse input response matrices [113].

##### Model-based RS

In contrast to neighbourhood-based methods, model-based RSs require an explicit model and a training process that determines the model parameters. Once learned, models are fast to apply to new data, and relatively little memory is needed to store model parameters. In addition, model-based RSs typically perform better in cases where the cell line-drug sensitivity matrix is sparse [113].

Different learning algorithms can be used to train parameters of model-based methods. Typical RS-based methods for DRP are matrix factorization methods that construct latent factor models [113]. Such methods commonly represent the cell line-drug sensitivity matrix as a product of two matrices, allowing an interpretation of cell lines and drugs as vectors in a latent space (Figure 3B).

Ammad-ud-din *et al.* [120] extended a kernelized Bayesian matrix factorization (KBMF) method that was originally developed for drug-protein interaction analysis [121], to combine different omics data and drug properties for DRP. Here, kernels allow capturing nonlinear relationships between drug response and both omics and drug features. To improve model interpretability, they further introduced component-wise MKL. In this method, biological pathway-based groups of features are used as model inputs, thus incorporating prior biological knowledge in the model [122].

Similarity-regularized matrix factorization by Wang *et al.* [123] employs regularization by GE-based cell line similarity and chemical feature-based drug similarity measures. More recently, a modified regularization term for an analogous model was proposed by Moughari and Eslahchi [124]. They showed that cell line similarities based on GE and drug similarity-based drug targets provide the best performance among a variety of cell line and drug features. To account for redundant information in similarities, the *WGRMF* method [125] approximates the cell line similarity and drug similarity matrix with a sparse matrix.

As the three above methods do not define any transformation matrix that projects cell lines and drug features into a latent space, they cannot predict drug sensitivity for new cell lines or drugs. Using a cell line projection matrix, Suphavilai *et al.* [126] developed a matrix factorization-based RS, which enables the prediction for new cell lines. In addition, Yang *et al.* [127] used a drug projection matrix that additionally enables the prediction for a new drug. They used a method called *MACAU* [128] to incorporate GE and drug target information to train parameters. Moreover, to make predictions for new cell lines, Emdadi & Eslahchi [129] employ a neighbourhood-based approach, in which the latent vector for the new cell line is calculated as the average latent vector of its neighbours. Their *DSPLMF* method trains a classification model based on logistic matrix factorization to predict the probability of a cell line being sensitive to a drug. Such models can be categorized as hybrid model-/neighbourhood-based *RS*. To further improve the performance of their method, they developed a feature selection method called *Auto-HMM-LMF* [130].

#### Network representation learning-based models

Network representation learning methods project networks into a low-dimensional space [131] which can be used as the input features for ML. Yang *et al.* [132] proposed a method, in which a response network is first constructed by combining cell line genetic aberrations, drug responses and a PPI network. In the response network, cell lines with similar drug responses are close to each other. Next, a graph representation learning algorithm is used to learn a projection of the cell lines from the response network to a space that preserves their neighbourhood proximity. Finally, an SVM is trained on the representation vector of cell lines for each drug to predict the drug response of cell lines. The idea behind this method is that the cell lines with similar neighbourhood topology profiles have similar drug responses. The DREMO method by Yu *et al.* [133] constructs two multilayer similarity networks for cell lines and drugs based on different molecular data types, and then diffusion component analysis [134] is used to represent the cell lines and drugs into low-dimensional feature vector spaces. Finally, the sensitivity of the cell lines to each drug is predicted using logistic regression.

#### Network diffusion-based methods

Zhang *et al.* [135] proposed a DRP method on the basis of network diffusion on a heterogeneous network, consisting of cell line similarities, drug similarities, target similarities, cell line-drug sensitivities and drug–target interactions. Their target similarity network is obtained by merging PPIs and co-expression networks. In this method, a diffusion algorithm, called information flow-based method [136], is used to infer unknown cell line-drug response to be sensitive on the basis of the known responses by considering the density of the connectivities associated with the cell lines sensitive to drugs.

#### Ensemble learning methods

Ensemble learning (introduced for SDL models in Section Ensemble Learning Methods) can also be applied for MDL models. Matlock *et al.* [137] used an ensemble of RF, KNN and ANN methods for DRP. Liu *et al.* integrated a low-rank matrix completion and a ridge regression model for DRP [138]. Sharma & Rani [139] proposed another ensemble learning approach incorporating KBMTL [140], STREAM [141], L21 [142] and multitask regression. They first trained four different regression methods for predicting drug responses and then combined the output of each model using weighted averaging. Su *et al.* [143] proposed the two-level *Meta-GDBP* method*.* The first level contains four independent predictive models, based on GE, chemical properties of drugs, biological processes (based on GO terms [144]) and biological pathways (based on KEGG pathways [145]). For each of these information sources, they evaluated the performances of elastic net and SVR predictors. The second-level model is a weighted linear combination of the outputs of the best-performing models in the first level.

## Evaluation of DRP models

Model evaluation, i.e. the assessment of the expected performance of a trained model on future data, needs to occur on test data that are independent from the training data. Otherwise, during training, the learned model parameters may be better adapted to the test data than could be expected for future data, thus leaving the test data evaluation optimistically biased. Data leakage, e.g. the—sometimes subtle—presence of information about the test data in the training data, may, for instance, occur when very similar data, such as drugs with similar structures, are present between training and test sets, or when the entire dataset is used for data preprocessing, e.g. for normalization across cell lines. There are different common ways of splitting a single dataset into training and test data [57]. In repeated random subsampling, the dataset is randomly split into training and test datasets repeatedly, typically, of given sizes. In each iteration, the model is trained on the training set and an evaluation metric (e.g. mean square error) is calculated on the test set. Finally, the model performance is estimated as the average evaluation metric over all iterations. In *k-*fold cross validation, the dataset is split into *k* subsets (folds, typically of identical size). In *k* iterations, each fold is used as a test set, and the remaining data are used for training. K-fold cross validation ensures that each data sample will appear in both train and test sets with the same frequency, and avoids stochastic fluctuations, particularly for small datasets. Moreover, in cases when *k*-fold cross validation would lead to training sets that are too small, typically, leave-one-out cross validation (LOOCV) is used, in which test sets of only a single sample are used in each iteration. It is worth noting that LOOCV is not recommended for large datasets since it is computationally expensive and leads to estimates of the model performance with high statistical variance [146]. Leave-pair-out cross validation (LPOCV) is a special case where only two samples are held out. Finally, there is a validation method specifically for RF called out-of-bag validation (refer to section Ensemble Learning Methods). In models with hyperparameters, an additional subset of the training data (validation set) is typically split-off from the training set before training to evaluate the model under different hyperparameter values before its version with the best hyperparameter is evaluated on the test set.

**Figure 4:**
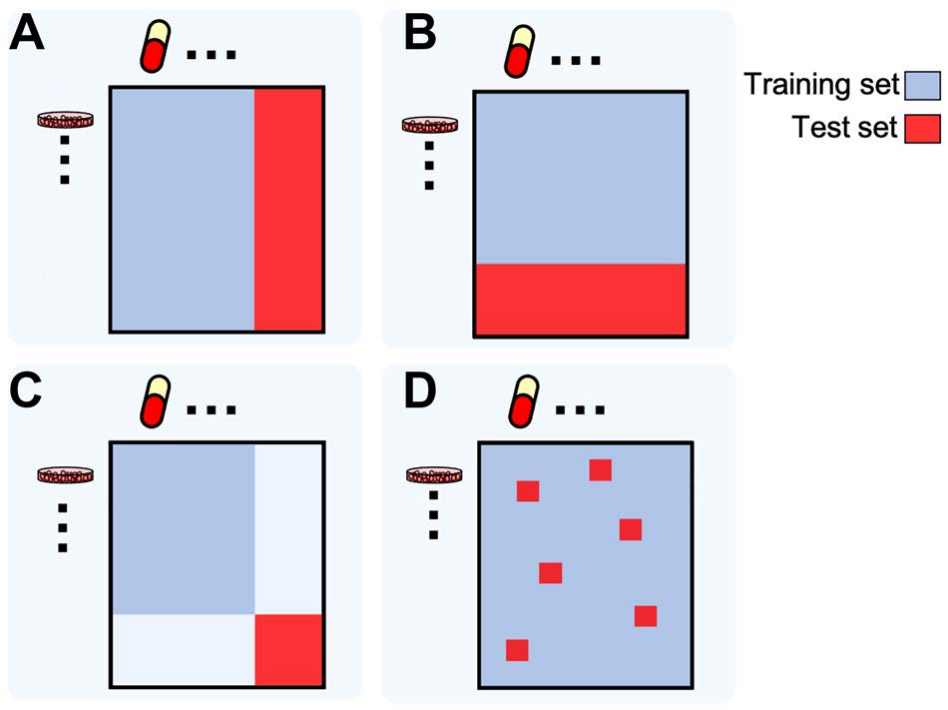
Different MDL model validation schemes. (A) Known cell line/new drug; (B) new cell line/known drug; (C) new cell line/new drug; (D) known cell line/known drug.

As the performance of MDL models for new data may depend on whether both drugs and cell lines are present or not in the training data (i.e. known or new), four different evaluation modes exist (Figure 4):

1. Known cell line/new drug*.* The prediction on new drugs typically uses known drug features. Leave-one-compound-out is a special case where only one drug is left out.

2. New cell line/known drug: This case is typical for personalized medicine applications. Leaving all cell lines derived from one tissue out as the test set is called leave-one-tissue-out.

3*.* New cell line/new drug: This evaluation corresponds to the most ambitious prediction task.

4. Known cell line/known drug: This evaluation corresponds to the least ambitious prediction task, which can be used to impute missing values.

Training and test sets can also be selected from different datasets. Cross-dataset evaluation thus also incorporates potential batch effects, i.e. systematically different characteristics between datasets, such as scale. In many real-world cases, heterogeneous output data present an additional challenge. For instance, regression models that predict IC50 values observed on cell lines may be difficult to evaluate against the drug effects as they are commonly characterized in clinical datasets.

In biomedical classification scenarios, the amount of available data may strongly vary across the different classes, which presents special challenges for learning and evaluation. To balance classes before learning, one can undersample the majority class, over-sample the minority class with replacement, or use synthetic data (e.g. SMOTE) [145, 146]. For a discussion of subtleties and pitfalls of these approaches, we refer the reader to the review by Chawla [147].

## Discussion and outlook

Machine learning is a subfield of artificial intelligence whose classification and prediction algorithms learn patterns underlying the data from examples. Omics data usable for DRP consists of different types of molecular profiles, typically from genomics, epigenomics, transcriptomics and proteomics. The different available data types capture different (potentially overlapping) facets of the biological drug response. Comparative studies found that, among different individual data types, GE enables the best predictive performance, which can then be improved only slightly by adding other data types [22, 24, 50, 148]. Iorio *et al.* [14] reported that GE is an individual data type that leads to the best prediction performance across a large set of cancers. Interestingly, they also show that the prediction performance of GE is highly correlated with tissue types [14]. However, employing tissue types along with GE as model input so far did not lead to performance improvements [40, 46, 50, 148]. In a study on myeloma, GE did not provide satisfactory results [149]. When considering a broader set of cancer types by themselves, genomic features appear to be the most informative single data type [14].

Simple linear regression methods are easily interpretable and have been used for drug-specific biomarker detection [16]; however, they cannot capture more complex associations in the data. On the other hand, the complexity of the output of many more advanced DRP ML methods—for instance, those based on many different transcripts—limits their use for the development of drug resistance biomarkers, and the extraction of new knowledge about the molecular mechanisms behind drug responses. We expect that ongoing improvements in the interpretation of ANNs [150, 151] will continue to be reflected in new approaches to DRP [65, 67].

DRP ML models can be categorized as single-drug and multi-drug models. MDLs can leverage data from multiple drugs, thus conferring robustness and the ability to predict responses to new drugs. MDL models have also been seen empirically to show better prediction performance than SDLs [94, 116, 126, 129], and to lead to more robust biomarkers [126]. Overall, RS and deep learning have been the most widely MDL methods used for DRP. The results reported in the literature suggest the superiority of RSs [24, 114, 116, 123, 124, 126, 129, 130] and deep learning methods [93, 96, 101, 104, 107] over more traditional approaches, such as linear regression, RF and SVM.

At this stage, statements about the current performance of ML-based DRP methods overall are difficult to make. In the clinic, molecular patient data are already an established input for treatment decisions [152], and the potential of ML-based DRP models to predict tumoral responses to anticancer drugs has been established [30, 44, 46, 58, 67, 71, 101]. However, ML-based DRP seems to be rarely used in clinical practice.

One major reason for the currently limited utility of DRP in the clinic may be the difficulty of addressing random and systematic variations between datasets, which is an issue in many applications of ML. Another reason may be the lack of interpretability of many DRP models in the context of other rich, and often unstructured, relevant additional clinical information that is currently not used by DRP models.

The future of drug prioritization in the clinic will undoubtedly be influenced by ML [153]. We believe that future improvements—especially, towards the use of DRP models in the clinic—will be catalyzed in a few distinct ways. Firstly, it can be expected that, as in other ML applications, larger systematic datasets will be able to drive performance increases, especially if new datasets are aimed at the more comprehensive characterization of the relevant biology. This is particularly true if one considers the current paucity of systematic data outside of the cell line context. Future datasets with the largest positive impact on DRP performance may therefore well come from advanced preclinical models. Although more, and more diverse, data can be thought to help in any ML approach, it is in particular deep learning, as a maximally flexible class of models, that is fundamentally capable of capturing the more subtle characteristics of larger datasets. The increasing availability of computing power—and computing as a part of the biomedical research enterprise—will likely enable the continued application of new, and computationally ambitious, ML technology to DRP.

A second, complementary, route towards higher performance of DRP models is the incorporation of prior knowledge. Until now, the incorporation of prior knowledge through biomolecular networks has led to only slight overall improvements at best [44, 49, 60]. However, Oskooei *et al.* [84] has found clear improvements for specific drugs, when additional information about drug targets was provided.

Various missing aspects of drug responses in the clinic may be addressable by either large datasets, or the incorporation of knowledge (or a combination of both), as long as various current systematic limitations of data and models are addressed. A first, major, systematic limitation of most DRP models is their focus on the short-term drug response in homogeneous cell populations. Neither the available cell line input data nor the learning approaches themselves currently address the complex evolutionary process that often leads to drug resistance in heterogeneous tumours [154]. Further development of clinically relevant machine learning-based DRP models may require the use of input data, such as genomic and transcriptomic single-cell data, that resolve the subclonal tumour structure [155], and explicit modelling of tumour evolution [156].

Another aspect of biological complexity that is typically not addressed by current data-driven approaches to DRP, or data, is the immune system, which plays a major part in many diseases, including cancer [157]. The state of the immune system in relevant tissue can be highly associated with chemotherapy resistance [158], and immunomodulators have, in the last decade, become major weapons themselves against cancer and other diseases [159]. The dbGap database [160] covers immune responses to drugs with RECIST parameters, and thus provides future opportunities for the incorporation of the immune system into DRP.

The microbiome is yet another complex part of human physiology known to regulate many aspects of human health, and, increasingly, it is also recognized as a mediator of drug effects [161, 162]. Evolving insight into the microbiome and its role in drug action may stimulate the adoption of the microbiome as part of DRP models [163], or lead to models in which the microbiome can then either act as an enhancer, or even become itself an active part of therapy through the molecules it secretes [164]. Related datasets, such as TCGA [25] microbiome data have, so far, not been included in DRP approaches.

Another area of potential future significance for machine learning-based DRP is the prediction of unwanted side effects or adverse drug reactions*.* Although methods for the prediction of more general adverse patient events currently appear to focus on electronic health records [165], increasing insight into the molecular basis of adverse drug reactions [166] and associated data may also permit the future integration with principles used today for DRP.

Eventually, DRP methods and their underlying principles may also be applicable to the study of environmental- or food-related molecules on human health. Perhaps spurred by the human genome as the exhaustive parts list of human biomolecules, environmental science has started to adopt comprehensive approaches to profile exogenous chemicals, and to study their impact on human health [167].

As the effects of environmental chemicals on human biology can, at some level, be considered as similar to drug effects, DRP principles and methods to predict the impact of drug combinations on human health may be transposable to the prediction of the effects of chemical mixtures, where, similarly to drug combinations, exhaustive testing of chemical mixtures is typically not possible. Analogously, the study of interactions between drugs and environmental and food-related chemicals [168] will have to rely on mechanistic insight and data-driven models increasingly obtained with the help of machine learning methods, such as those developed for DRP.

**Box I. Common Preclinical drug response metrics**

Several common drug sensitivity metrics are used in DRP input data. All are obtained from sigmoidal dose–response curves that record cell viability (the activity of a specific biological process, or the number of cells) across a range of drug concentrations. Drug sensitivity is commonly determined relative to two reference values: *control* (i.e. cell viability in the absence of the drug) and maximal *response* (the maximally observed difference of cell viability, relative to control)*.* Based on these two concepts, pharmacogenomic databases use a variety of metrics to quantify growth inhibition [169, 170], the most important being:

- IC50 (inhibition concentration of 50%): the drug concentration at which cell viability is reduced to the half of control.
- GI50 (growth inhibition of 50%): the drug concentration that reduces growth by half.
- EC50 (effective concentration of 50%): the drug concentration that causes the half of the maximal response. Also called ‘relative IC50’.
- AUC (area under the dose–response curve): the area under the dose–response curve.
- AA (Activity *area*): the area over the dose–response curve.

It is worth noting that the cell lines that are sensitive to a drug will get lower IC50, GI50, EC50 and AUC values, and higher AA value.

**Key Points**

- Biological knowledge can be leveraged for machine learning of drug response prediction, e.g. through biomolecular networks.
- Recent advances in machine learning, e.g. in the areas of deep learning and multitask learning, have translated into increased performance of drug response prediction.
- Large-scale pharmacogenomic datasets for advanced preclinical models are urgently needed for the future development of data-driven drug response prediction.

## Notes

**Farzaneh Firoozbakht** is a researcher in the Systems Biology Group at Institut Pasteur, Paris, France.

**Behnam Yousefi** is a PhD student in the Systems Biology Group at Institut Pasteur, Paris, France, and Sorbonne Université, École Doctorale Complexité du vivant, Paris, France.

**Benno Schwikowski** is a Research Director and Group Leader of the Systems Biology Group at Institut Pasteur, Paris, France.
